# MicroRNA-506 is up-regulated in the development of pancreatic ductal adenocarcinoma and is associated with attenuated disease progression

**DOI:** 10.1186/s40880-016-0128-9

**Published:** 2016-07-02

**Authors:** Run-Fen Cheng, Jian Wang, Jing-Yi Zhang, Lin Sun, Yan-Rui Zhao, Zhi-Qiang Qiu, Bao-Cun Sun, Yan Sun

**Affiliations:** Department of Pathology, Tianjin Medical University Cancer Institute and Hospital, Tianjin, 300060 P. R. China; Department of Pancreatic Oncology, Tianjin Medical University Cancer Institute and Hospital, Tianjin, 300060 P. R. China; Department of Epidemiology and Biostatistics, Tianjin Medical University Cancer Institute and Hospital, Tianjin, 300060 P. R. China; Department of Pathology, Tianjin Medical University, Tianjin, 300070 P. R. China

**Keywords:** Pancreatic ductal adenocarcinoma, miR-506, Tumor suppressor, Development, Progression

## Abstract

**Background:**

MicroRNA-506 (miR-506) has been reported to function in several tumors as a tumor suppressor gene or oncogene. However, the expression and role of miR-506 in pancreatic ductal adenocarcinoma (PDAC) remains unclear. In this study, we aimed to evaluate the phenotype of miR-506 in PDAC.

**Methods:**

Using miRNA in situ hybridization, we examined the expression of miR-506 in 113 PDACs and 87 paired normal pancreatic tissues. We evaluated miR-506 expression in PDAC cells, normal pancreatic ducts, and acinus/islands, and we analyzed the associations between miR-506 expression and the clinicopathologic characteristics of PDAC patients.

**Results:**

miR-506 expression was higher in PDAC than in matched normal pancreatic ductal cells (*P* < 0.001). On the other hand, the combined group of well and moderately differentiated PDACs showed higher levels of miR-506 than the poorly differentiated ones (*P* = 0.023). Moreover, miR-506 expression was negatively associated with pathologic T category (*P* = 0.004) and lymph node metastasis (*P* = 0.033), suggesting that miR-506 might inhibit the progression of PDAC.

**Conclusions:**

Our results suggest that miR-506 either plays a role as an oncogene in the tumorigenesis and a tumor suppressor in the progression or serves as a house-keeping, tumor-suppressing miRNA, whose expression can be activated by oncogenic signals in early development to hinder the progression of PDAC.

## Background

Pancreatic ductal adenocarcinoma (PDAC) is a lethal malignancy, with a median patient survival of 6 months and a 5-year overall survival rate of 5% [1, 2]. The high rate of lethality from PDAC is primarily due to the advanced stage of disease at diagnosis, which precludes curative surgery and leads to a poor prognosis. Therefore, to improve patient survival, early diagnosis of PDAC is extremely important. Routine imaging methods, such as ultrasound, computed tomography, and magnetic resonance imaging, are not sensitive enough in early detection of PDAC [3]. Although carbohydrate antigen 19-9 (CA19-9) is considered the most useful biomarker in the early detection and prognostic assessment of PDAC, the sensitivity and specificity of circulating CA19-9 detection is still poor [4, 5]. At present, treatment options for patients with PDAC are limited. Gemcitabine-based chemotherapy remains the standard of care for patients with advanced PDAC, but intrinsic and acquired chemoresistance is common [6]. The epithelial growth factor receptor inhibitor erlotinib is the only targeted agent currently used in the treatment of PDCA, but only a fraction of PDAC patients can benefit from it [7]. Therefore, to develop more efficient diagnostic and therapeutic strategies for PDAC, we need to elucidate the molecular mechanisms underlying its development and progression.

MicroRNAs (miRNAs) are small non-coding RNAs that inhibit the expression of target genes by base pairing to complementary sites in the 3′-untranslated region of target mRNAs, leading to translational repression or the degradation of the target mRNAs [8]. Recent studies have shown that miRNAs elicit oncogenic or tumor-suppressive functions by directly targeting oncogenes or tumor suppressor genes, respectively [8–10]. Deregulated miRNAs have been found to play pivotal roles in PDAC development and progression by affecting multiple cellular processes, such as cell proliferation, apoptosis, survival, invasion, metastasis, and chemotherapeutic resistance of PDAC [11].

miR-506, located on Xq27.3, was identified as a member of the miR-506-514 cluster (including miR-506, miR-507, miR-508, miR-509, miR-510, miR-513 and miR-514) and was preferentially expressed in the testes of primates [12]. miR-506 was shown to inhibit tumor progression by suppressing epithelial-to-mesenchymal transition (EMT), cell migration and invasion, cell proliferation, and angiogenesis and by promoting cell senescence in ovarian, gastric, breast, colorectal, and liver cancers as well as glioma [13–26]. In contrast, the miR-506-514 cluster was shown to be an activator in initiating tumor transformation in malignant melanoma [27]. In pancreatic cancer cells, miR-506 was shown to inhibit cell proliferation by targeting Pim-3 proto-oncogene (*PIM3*), a member of the proto-oncogene PIM family [28]. Using real-time reverse transcription-polymerase chain reaction (qRT-PCR), Du et al. [28] examined the expression of miR-506 in 38 pancreatic cancers and matched adjacent normal tissues and found that 71% (27/38) of cases exhibited down-regulated miR-506 expression.

In this study, using miRNA in situ hybridization (ISH), we examined the expression of miR-506 in 113 PDAC and 87 matched normal pancreatic tissues. Considering the complicated constituents of pancreatic tissue, we believed that miRNA ISH that can be linked to morphology is a more accurate option to demonstrate miR-506 expression in specific cells. We evaluated the expression of miR-506 in PDAC cells, normal pancreatic ductal cells, acinar cells, and islands. In addition, we analyzed the relationship between miR-506 expression and clinicopathologic parameters to elucidate the role of miR-506 in the progression of PDAC.

## Methods

### Patients and tissue samples

We collected 200 formalin-fixed, paraffin-embedded pancreatic tissue samples (including 113 cases of tumor tissue and 87 cases of non-tumor tissue in the resection margin) from the Department of Pathology at Tianjin Medical University Cancer Institute and Hospital (Tianjin, China). All samples were from 113 patients with PDAC who underwent surgical operation between 2007 and 2010, and none of the patients received any preoperative chemotherapy or radiotherapy. All specimens and clinical data were collected after our study received approval from Institutional Review Board of Tianjin Medical University Cancer Institute and Hospital.

### Tissue microarray construction

Tissue microarrays (TMAs) were constructed using a manual tissue microarray instrument (Beecher Instruments, Sun Prairie, WI, USA) equipped with a 2.0-mm punch needle, as described previously [29]. Five TMA blocks were prepared for the 200 pancreatic tissue specimens. For each tissue sample, the typical area was selected based on the appearance of the original hematoxylin and eosin-stained slides. For the non-tumor tissue in surgical resection, we chose the typical areas with normal pancreatic ducts. For the 87 patients with both PDAC and non-tumor tissues, the matched tumor and non-tumor tissues were placed in adjacent cores.

### miRNA ISH

The 4-μm paraffin-embedded TMA sections were hybridized with the double-DIG-labelled miRCURY LNATM detection probe, hsa-miR-506 (38314-15, Exiqon, Woburn, MA, USA) for 2 h at 55°C, as described previously [13, 14]. The digoxigenins were detected with a polyclonal anti-DIG antibody and an alkaline phosphatase-conjugated second antibody (Ventana, Tucson, AZ, USA), using Nitroblue tetrazolium-5-bromo-4-chloro-3-indolyl phosphate (NBT-BCIP) as the substrate. The LNA U6 snRNA probe (Exiqon, Woburn, MA, USA) was used as a positive control for every TMA core, with blue staining in the nucleus. For miR-506, blue staining in the cytoplasm was defined as positive signals. Signals in tumor cells and pancreatic non-tumor tissue (pancreatic ducts, acinar cells, and islands) were quantified by two senior pathologists, using a previously described scoring system [13, 14] with some modifications. The signal intensity (0, no signal; 1, weak signal; 2, intermediate signal; and 3, strong signal) and the percentage of positive cells (0, 0%; 1, <25%; 2, 25%–50%; and 3, >50%) were multiplied to obtain a score (0, 1, 2, 3, 4, 6, or 9). Low and high miR-506 expression levels were defined as scores of <4 and ≥4, respectively.

### Statistical analysis

Statistical analyses were performed using GraphPad Prism 5.0 (GraphPad Software, Inc., La Jolla, CA, USA) and SPSS version 17.0 softwares (SPSS Inc., Chicago, IL, USA). Student’s t test and the Chi square test were used to compare miR-506 levels between different groups. All statistical tests were two-sided, and *P* values less than 0.05 were considered statistically significant.

## Results

### Patient characteristics

Of the 113 patients with PDAC, 64 (56.6%) were men, and 49 (43.4%) were women. Patient age ranged from 31 to 79 (median, 59) years. Fifty-two (46.0%) tumors were found in the head, 33 (29.2%) in the uncinate process, and 28 (24.8%) in the body and tail of the pancreas. Of the 113 PDAC patients, 79 (69.9%) underwent pancreaticoduodenectomy, 30 (26.5%) distal pancreatectomy plus splenectomy, 2 (1.8%) pancreaticoduodenectomy plus portal vein replacement, 1 (0.9%) total pancreas resection, and 1 (0.9%) pylorus-preserving pancreaticoduodenectomy. The largest tumor diameter ranged from 1 to 10 cm (4.2 ± 1.8 cm). Histopathologically, 10 (8.9%), 59 (52.2%) and 44 (38.9%) PDACs were well, moderately, and poorly differentiated, respectively. Twenty-eight (24.8%) PDACs were limited to the pancreas (pathologic T category (pT1 and pT2), whereas 85 (75.2%) PDACs invaded adjacent organs or spread to the abdominal cavity (pT3 and pT4). Except the 15 patients for whom not enough information was available to evaluate the status of lymph node metastasis, lymph node metastasis was found in 21 (18.6%) patients, and 77 (68.1%) patients had no lymph node metastasis.

### miR-506 expression was up-regulated in PDAC compared with normal pancreatic ducts

**Fig. 1:**
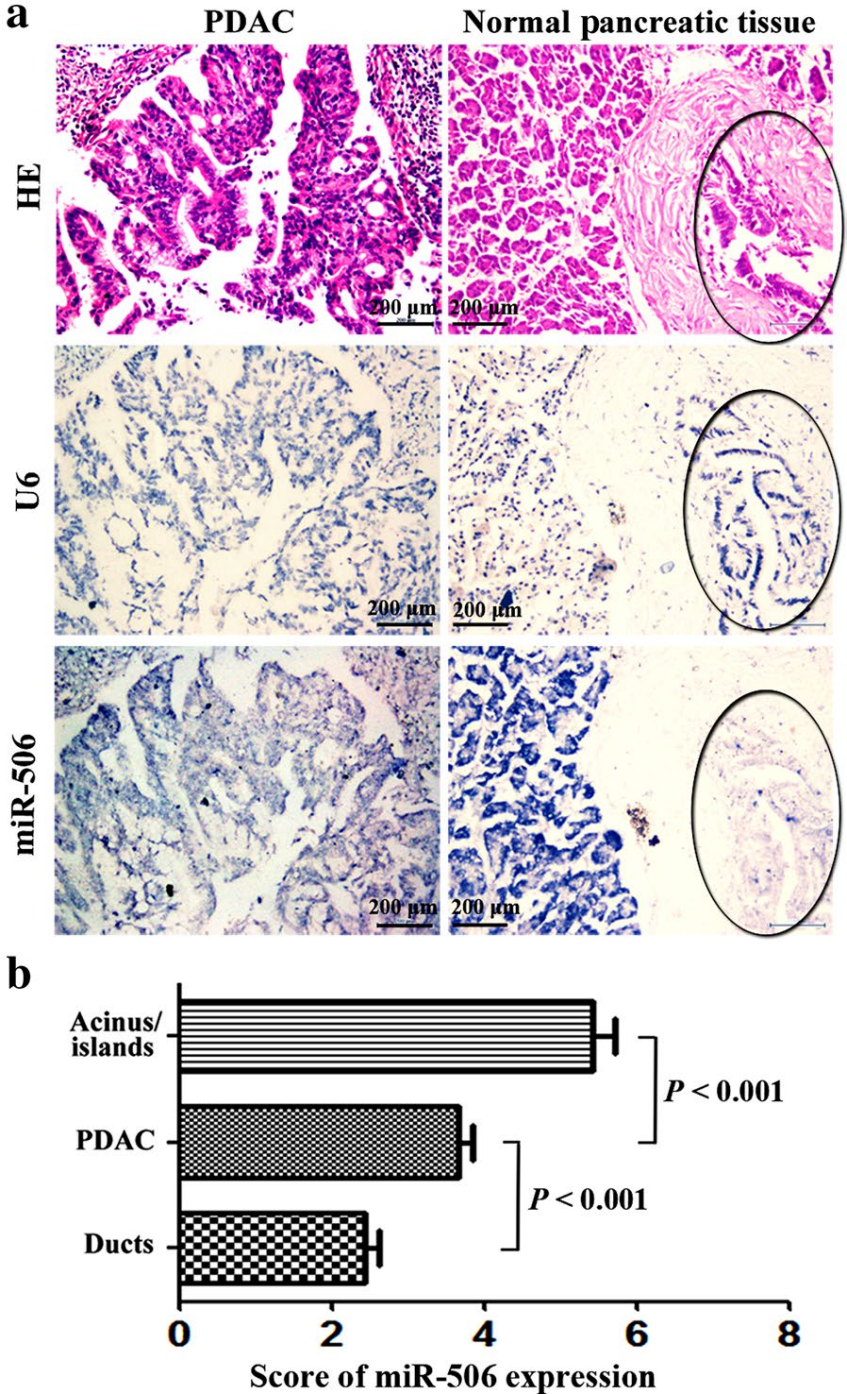
The expression of miR-506 in pancreatic ductal adenocarcinoma (PDAC) and matched normal pancreatic tissues. **a** Representative images of hematoxylin and eosin (HE) staining, U6 in situ hybridization (ISH) (positive control), and miR-506 ISH. The *circles* indicate normal pancreatic ducts. **b** Statistical analysis showed that miR-506 expression in PDACs is significantly higher than that in normal pancreatic ducts but lower than that in normal pancreatic acinus/islands

Pancreatic lobules are composed of ducts, acinus and pancreatic islands. PDAC mainly originates from ductal cells. We specifically analyzed the miR-506 expression in PDAC and normal pancreatic ducts. Of the 113 PDACs analyzed on TMAs, three were not evaluable owing to tissue shedding, insufficient tumor cells, or invalid staining of the positive control (U6). Of the 87 pancreatic non-tumor tissues analyzed on TMAs, 75 and 69 were identified to have normal pancreatic ducts and acinus/islands, respectively. The results of ISH showed that miR-506 expression was higher in PDAC than in the matched normal pancreatic ducts (Fig. 1a). Statistical analysis showed significant differences in all evaluable samples (*P* < 0.001, Fig. 1b). On the other hand, miR-506 expression was significantly lower in PDAC than in normal pancreatic acinus/islands (*P* < 0.001; Fig. 1).

### PDAC showed a higher miR-506 expression compared with pancreatic intraepithelial neoplasia

**Fig. 2:**
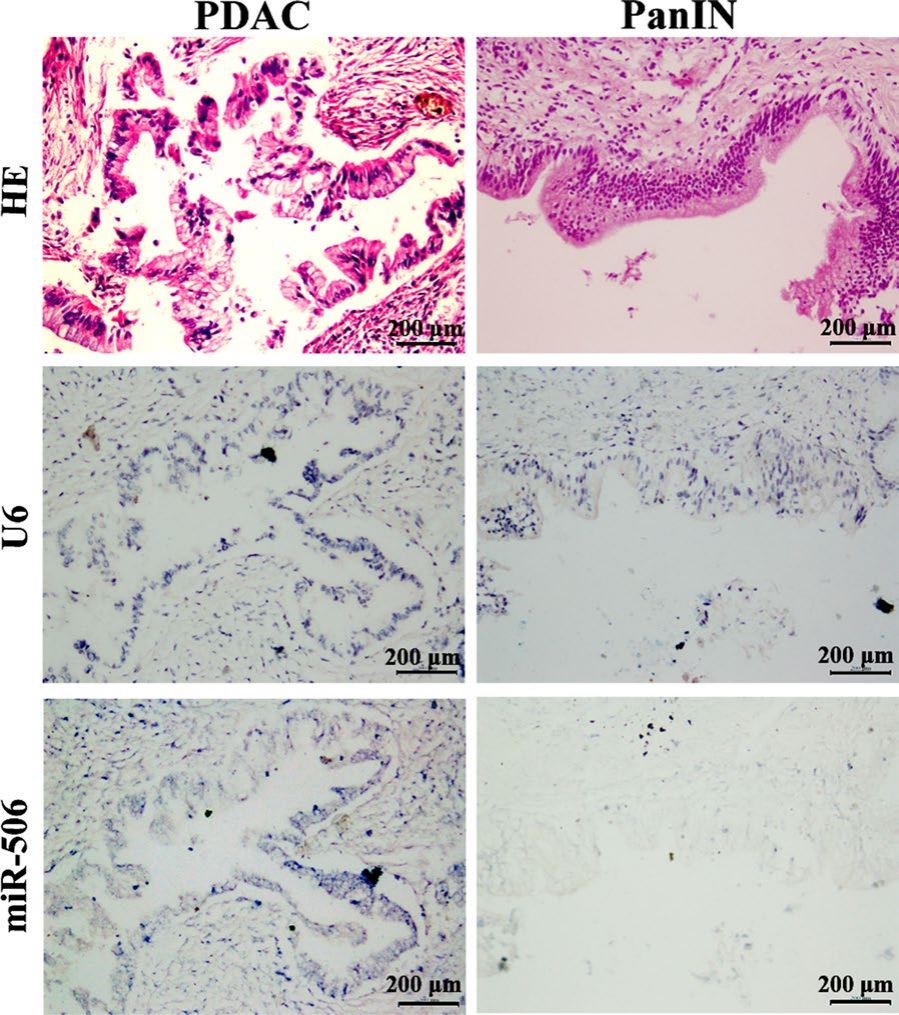
The expression of miR-506 in one PDAC and matched low-grade pancreatic intraepithelial neoplasia (PanIN). miR-506 expression is obviously higher in PDAC than in the matched low-grade PanIN

Pancreatic intraepithelial neoplasia (PanIN) is a precursor lesion of PDAC [30, 31]. PDAC is usually accompanied by PanIN [32]. On TMA, we identified three PanINs together with PDAC or in non-tumor tissue. ISH results showed that, in all three patients, miR-506 expression was higher in PDAC than in PanIN, although the limited number of patients precluded statistical evaluation (Fig. 2).

### Associations between miR-506 expression and clinicopathologic parameters

**Fig. 3:**
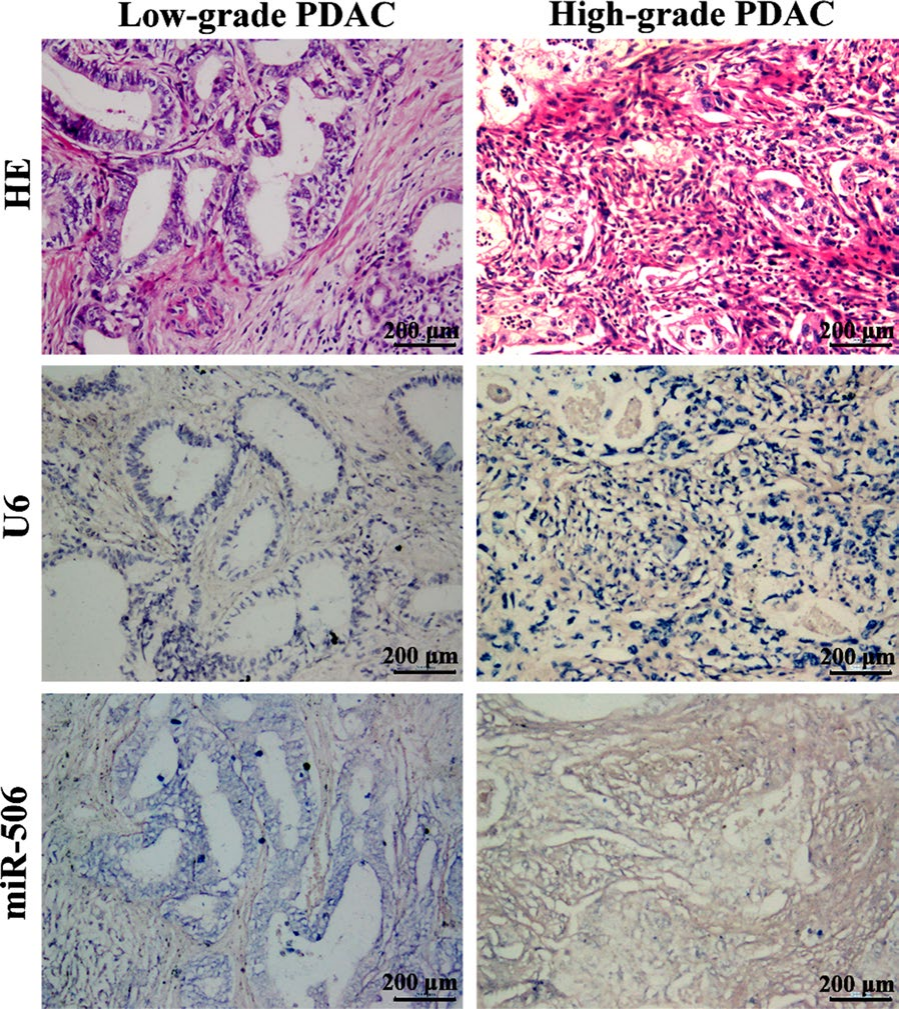
The expression of miR-506 in low-grade (well and moderately differentiated) and high-grade (poorly differentiated) PDACs. miR-506 expression is obviously higher in low-grade PDACs than in high-grade ones

We further analyzed the relationship between miR-506 expression and clinicopathologic parameters in the 110 PDAC patients with available miR-506 ISH results. miR-506 expression was relatively stronger in well and moderately differentiated PDACs compared with poorly differentiated PDACs (Fig. 3). Statistical analysis revealed that the rate of high miR-506 expression in well and moderately differentiated PDACs was higher than that in poorly differentiated ones (*P* = 0.023; Table 1). Moreover, the rates of high miR-506 expression in the pT1-T2 and N0 groups were significantly higher than those in the pT3-T4 (*P* = 0.004) and N1 groups (*P* = 0.033; Table 1), respectively. We found no association between miR-506 expression and age, sex, or tumor site and size (Table 1).

**Table 1:** The relationship between miR-506 expression and clinicopathologic parameters in 110 PDACs

Variable	No. of patients	miR-506 expression [case (%)]		χ^2^	*P* value
		Low	High		
Age (years)					
≤59	57	41 (71.9)	16 (28.1)	2.194	0.139
>59	53	31 (58.5)	22 (41.5)		
Sex					
Men	63	45 (71.4)	18 (28.6)	2.327	0.127
Women	47	27 (57.4)	20 (42.6)		
Tumor site					
Head	51	35 (68.6)	16 (31.4)	1.551	0.461
Uncinate process	32	22 (68.8)	10 (31.2)		
Body and tail	27	15 (55.6)	12 (44.4)		
Tumor size (cm)					
≤4	67	45 (67.2)	22 (32.8)	0.222	0.638
>4	43	27 (62.8)	16 (37.2)		
Histologic grade					
G1, G2	68	39 (57.4)	29 (42.6)	5.170	0.023
G3	42	33 (78.6)	9 (21.4)		
pT category					
T1, T2	28	12 (42.9)	16 (57.1)	8.482	0.004
T3, T4	82	60 (73.2)	22 (26.8)		
Lymph node metastasis^a^					
No	74	45 (60.8)	29 (39.2)	4.542	0.033
Yes	21	18 (85.7)	3 (14.3)		

^a^No information about lymph node metastasis in 15 patients

## Discussion

In this study, we demonstrated that miR-506 was up-regulated in PDACs compared with normal pancreatic ducts. Du et al. [28] examined miR-506 expression in 38 PDAC patients with RT-PCR and showed that miR-506 was significantly down-regulated in PDACs compared with normal adjacent tissues. The reasons for the inconsistency may be owing to differences in the sample size and the testing method. One of the advantages of miRNA ISH is the combination of the morphology and the expression of miRNAs. The structure of the pancreas is complex and includes pancreatic ducts, acinus, and islands. Moreover, in normal pancreatic lobules, there are many more acinar cells than ductal cells. We think that, because most PDACs originate from ductal cells [33], comparing the expression of miR-506 in PDAC cells with that in normal pancreatic ductal cells to evaluate the role of miR-506 in PDAC is more accurate. Furthermore, one typical feature of PDAC is the obvious desmoplastic response. In addition, PDACs often have obscure boundaries, and the tumor cells usually invade into adjacent pancreatic acinus cells and pancreatic islands. Therefore, it is important to distinguish tumor cells from stromal cells and differentiate pancreatic ductal cells from the surrounding acinus and islands. The results of qRT-PCR are for the whole cells that were used for RNA extraction. If we want to evaluate the expression of specific cells, laser capture microdissection is suggested before RNA extraction and qRT-PCR. However, miRNA ISH has its disadvantages, such as it not being a quantitative method. To mitigate the disadvantage, we used a semi-quantitative scoring system, as described in the Methods section, and performed the analysis in a relatively large patient population. Further studies are warranted to identify the expression of miR-506 in PDAC.

Increasing evidence has demonstrated that PanIN is the most direct precursor lesion of PDAC [34]. Studies have shown that patients with benign pancreatic tumors whose surgery margin had high-grade PanIN were diagnosed with PDAC several months or several years after the surgery [35–37]. The molecular alteration is accumulated during the progression from PanIN to PDAC [38]. Kirsten ras oncogene (*KRAS*) mutation was found in 36% of PanIN1A cases, 44% of PanIN1B cases, 87% of PanIN2/3 cases, and more than 90% of PDACs [35, 39]; therefore, *KRAS* mutation was considered an early genetic alteration event of PDAC [40]. Cyclin-dependent kinase inhibitor 2A (*CDKN2A*) mutation can be found in PanIN2, and mutations of tumor protein p53 (*TP53*), breast cancer 2 (*BRCA2*), and SMAD family member 4 (*SMAD4*) were found mainly in PanIN3 or PDAC, which were recognized as late events of PDAC initiation [41, 42]. In our study, because of the limitation of TMA, we found only three low-grade PanINs in the non-tumor tissue or among tumor cells. However, in all three cases, miR-506 expression was higher in PDAC than in PanIN. Considering that miR-506 was up-regulated in PDAC compared with normal pancreatic ducts and low-grade PanIN, miR-506 may play a role as an oncogene in the late phase of PDAC initiation. Alternatively, the activation of miR-506 expression may be a result of feedback response of the tumor suppression system to oncogenic signaling. For house-keeping tumor suppressor genes such as *TP53*, such an oncogene-activated elevation is an important mechanism [43, 44]. Future studies will be needed to differentiate these two scenarios.

miR-506 was shown to inhibit EMT by targeting snail family transcriptional repressor 2 (*SNAI2*), vimentin (*VIM*), cadherin 2 (*CDH2*), CD151 molecule (*CD151*), ETS proto-oncogene 1 (ETS1), and enhancer of zeste 2 polycomb repressive complex 2 subunit (*EZH2*) in ovarian, breast, gastric, and colorectal cancers [13, 14, 17, 45, 46]. In our study, miR-506 expression was significantly higher in well and moderately differentiated PDACs than in poorly differentiated ones. Compared with well and moderately differentiated tumors, the tumor glands in poorly differentiated PDACs are more irregular; they are even replaced with solid nests or trabeculae. Consequently, the expression of E-cadherin and cell adhesion in cancer cells is lower in poorly differentiated PDACs than in well and moderately differentiated ones [47, 48], which was consistent with our finding of miR-506 down-regulation in poorly differentiated PDACs. Further studies will be needed to evaluate cause and effect between miR-506 expression and tumor differentiation.

Invasion and metastasis were involved in tumor progression. According to the TNM staging system of PDAC [49], the cancer cells at pT3 and pT4 stages (beyond the pancreas) are more invasive than those at pT1 and pT2 stages (limited to the pancreas). Our data showed that miR-506 expression was lower in cancer cells at pT3 and pT4 stages than in those at pT1 and pT2 stages. Furthermore, we found lower levels of miR-506 in the PDACs with lymph node metastasis than in those without metastasis. Therefore, miR-506 is likely to function as a tumor suppressor to attenuate progression of PDAC.

In summary, by using miRNA ISH in a large-scale study of PDAC and normal pancreatic tissues, we demonstrated that miR-506 was up-regulated in PDAC compared with normal pancreatic ducts. On the other hand, miR-506 expression was negatively associated with pT stage and lymph node metastasis, suggesting that miR-506 might inhibit the de-differentiation and invasion of PDAC cells as a tumor suppressor. The bimodal pattern of miR-506 expression in different phases of PDAC suggests that miR-506 either plays a role as an oncogene in the tumorigenesis and a tumor suppressor in the progression or serves as a house-keeping, tumor-suppressing miRNA, whose expression can be activated by oncogenic signals in early development to hinder the progression of PDAC. Further studies are needed to gain deeper insights into the role and mechanism of miR-506 in PDAC.
